# Influencers on quality of life as reported by people living with dementia in long-term care: a descriptive exploratory approach

**DOI:** 10.1186/s12877-015-0050-z

**Published:** 2015-04-23

**Authors:** Wendy Moyle, Deirdre Fetherstonhaugh, Melissa Greben, Elizabeth Beattie, AusQoL group

**Affiliations:** Director Centre for Health Practice Innovation, Menzies Health Institute Queensland, Griffith University, Nathan Campus, Brisbane, Australia; Dementia Collaborative Research Centre-Consumers and Carers (DCRC-CC), Queensland University of Technology, Brisbane, Australia; Australian Centre for Evidence Based Aged Care (ACEBAC), La Trobe University, Melbourne, Australia; Research Assistant, Centre for Health Practice Innovation, Griffith University, Brisbane, Queensland Australia; Director DCRC-CC, Queensland University of Technology, Brisbane, Australia

**Keywords:** Dementia, Quality of life, Long-term care, Qualitative research

## Abstract

**Background:**

Over half of the residents in long-term care have a diagnosis of dementia. Maintaining quality of life is important, as there is no cure for dementia. Quality of life may be used as a benchmark for caregiving, and can help to enhance respect for the person with dementia and to improve care provision. The purpose of this study was to describe quality of life as reported by people living with dementia in long-term care in terms of the influencers of, as well as the strategies needed, to improve quality of life.

**Methods:**

A descriptive exploratory approach. A subsample of twelve residents across two Australian states from a national quantitative study on quality of life was interviewed. Data were analysed thematically from a realist perspective. The approach to the thematic analysis was inductive and data-driven.

**Results:**

Three themes emerged in relation to influencers and strategies related to quality of life: (a) maintaining independence, (b) having something to do, and (c) the importance of social interaction.

**Conclusions:**

The findings highlight the importance of understanding individual resident needs and consideration of the complexity of living in large group living situations, in particular in regard to resident decision-making.

## Background

Approximately 35.6 million people globally [1] including over 300,000 Australians are living with dementia [2]. Alongside the ageing of the population it is expected that this number will double every 20 years [1]. People with dementia live in the community until diminishing cognition, concurrent physical decline, and changes in behaviour over time often mean that they may need more care than is possible at home and this may result in them entering permanent long-term care. This expected increase in the number of people living with dementia will result in a higher proportion of people with dementia living in long-term care (LTC) in the years to come. It is estimated that currently over 50% of LTC participants in Australia have dementia [3].

The Australian LTC environment has improved over the last two decades as care homes are required to be accredited to receive Australian Government subsidies [4] and care providers have also been encouraged to implement person-centred approaches to care that reflect importance of respect for dignity, worth and human rights [5]. Alongside these improvements in Australia there has been a global move towards understanding the effect of care provision on people living with dementia and the influence of care on this population’s quality of life (QOL) [6,7]. Maintaining a good QOL of this population should be an imperative as there is currently no cure for dementia. Although definitions of QOL vary, it is widely acknowledged that the person with dementia may be the best informant when it comes to measuring their QOL [8]. Quality of life is something that one has, or possesses, and in the case of care provision QOL may be used as a benchmark for caregiving. Understanding QOL from the perspective of the person with dementia can help to enhance respect for the individual and to improve care provision.

The research literature suggests that measuring QOL can be challenging, in particular in the later stages of dementia [9]. Cognitive impairment that affects memory, attention, language and insight impacts on the individual’s ability to comprehend and communicate their subjective view of QOL [8,10]. However, there are many examples where people with mild to moderate dementia [11,12] and some with severe dementia (e.g. [13,14]) have been able to give meaningful assessments of their QOL. We know also that people with dementia rate their QOL higher than proxy reports [15,16]. Lack of communication between the proxy and the person with dementia, and perceived dependency and incompetence of the person with dementia are factors that have been identified as contributing to differences in ratings between proxies and residents [17]. In addition recent research has shown that the factors that influence QOL include relationships, feeling valued in society and having control over things in life [18]. We do not know, however, what people with dementia would like to improve in their life, in particular when they live in LTC. Nor do we know about the strategies that need to be put into place in LTC to ensure that people with dementia can experience the things that they perceive will make their life worth living. Therefore the aim of this paper is to understand the perception of QOL of older people living with dementia in long-term care and more importantly, the influences on their QOL and strategies to improve QOL.

This paper outlines the findings from the qualitative component of a larger Australian national study [19]. The main study recruited 480 participants and their family members from 53 facilities (approximately 10 per facility) in six Australian States and one Territory. The larger mixed methods study used a sequential design where the qualitative phase followed the quantitative phase of data collection. In this type of research design the quantitative and qualitative data are related to each other in that they address the same questions – one builds on the other. The qualitative phase was used to help us to understand, or to address, our original research questions about QOL for this population. These questions were: What is the QOL experienced by people with dementia residing in long-term care in Australia? What are the important issues relating to QOL from the perspective of people with dementia? Are there any facility-level characteristics associated with QOL for people with dementia?

## Methods

A descriptive exploratory qualitative research design was used and included a case study approach, which gave us the flexibility to include as much variation in each of the cases as possible. A case study approach allows the exploration of individuals as well as organisations and can also help researchers to develop interventions [20]. For efficiency we drew a subsample from the larger sample of participants from two Australian states.

Queensland University of Technology Human Research Ethics Board approved the study. Participants were informed in writing as well as by spoken word about the purpose of the study and in line with the ethics approval where possible they, or their proxies gave written consent for participation. Oral accent to participate in an interview was also sought from participants at the time of the interview.

### Participants

Participants screened for eligibility in the larger study from two Brisbane and two Melbourne participating facilities were invited to participate. Inclusion criteria: (1) resident of an aged care facility in a subsample of four facilities, (2) resided in their current facility for at least 3 months, (3) aged over 65, and (4) with a recorded diagnosis of dementia. Exclusion criteria: Resident with a diagnosis of Huntington’s Disease, Parkinson’s Disease, or Alcohol Related Dementia. A purposive sample of n = 12 (3 people per site (6 per State) allowed variation in gender, age, culture, stage of disease and variation in facilities, and public/non-profit/private facilities. We also took the view that if more than 12 participants were needed to reach data saturation we would recruit additional participants during the interview stage.

### Data source

Individual face-to-face interviews with 12 people living with dementia provided the primary source of data. Two interviewers experienced in dementia and qualitative methods conducted the interviews. The interviewers were trained by the team in how to conduct the interview questions and practice sessions were used to ensure consistency in the approach. The interview schedule examined QOL and was organised around five areas of investigation: 1. *Meaning in life, 2. Physical factors, 3. Psychological factors, 4. Social factors* and 5.*Psychological factors*, related to QOL. An expert panel shaped the areas of investigation and the questions based on the study’s research questions, the panel’s expertise, literature, and interview data that had already been obtained from families. The interview questions asked participants to tell the interviewer about:

1. The things in your life that help to make your life meaningful?
2. Your description of quality of life?
3. The physical things (self and environment) that help to make your life meaningful?
4. The social things that help to make your life meaningful?
5. The way you spend your time each day?
6. The psychological factors in the nursing home that help to make your life meaningful?
7. The things you would like to change in your day to help make your life worth living?
8. The physical things (self and environment) that need to be changed to make your life worth living.
9. The social things that need to be changed to make your life worth living.
10. The psychological things that need to be changed to make your life worth living.

We evaluated the data after each interview to determine if new insights were being produced from each interview. Rich data with thick description consistent with data saturation was achieved after interviews with nine participants. We conducted an additional three interviews and found that no new insights occurred in these interviews suggesting that we had established data saturation.

### Data analysis

Interviews were transcribed verbatim from audio recordings and then checked for accuracy. The study focused on QOL as experienced and described by people with dementia. Therefore, although participants sometimes discussed a range of material, the analysis focused only on the data that was relevant to understanding QOL [21]. Data were analysed thematically from a realist perspective [21]. A realist perspective reports “experiences, meanings and the reality of the participants” ([21], p.81) in a way that is value free. This is in contrast to a constructionist approach, which examines the events and the effects of the discourses. The approach to the thematic analysis was inductive and data-driven and followed the steps outlined by Braun and Clarke [21]: familiarisation with the data through repeated reading of the transcripts, systematically generating initial codes across the entire data set, identifying themes among the generated codes, reviewing themes to check internal and external validity, and defining and naming themes. Codes were initially assigned by one team member (MG) and then discussed and compared with another (WM). A further team member (DF) reviewed the data to verify the findings and the credibility of the findings in relation to whether they were believable and could be applied to the context. Atlas.ti 7.1.6 software [22] was used for data management and coding.

## Results

Participants included nine women and three men (age range 73–96 years; time living in the facility seven months-seven years and two months). All participants had a diagnosis of dementia with the majority having Alzheimer’s disease (AD) (see Table 1).

**Table 1:** **Participant demographics and characteristics**

**Gender & code**	**Age**	**MMSE score**	**Diagnosis**	**Time in facility**	**Mobility**	**Main comorbidities**	**Visitors (frequency of visits)**
Male R1	93	17	AD	11 months	Assisted transferring & locomotion	Confusion, Angina, Hypertension	Wife (daily); daughter (weekly)
Female R2	96	24	Unspecified dementia	7 y, 2 m	Supervised transferring & locomotion	Hearing impairment & cataracts	Daughter (weekly)
Male R3	95	17	Unspecified dementia	1y, 7 m	Assisted transferring & locomotion	Depression, anxiety, deafness & confusion	Son (daily)
Female R4	83	22	VD	9 m	Assisted transferring & locomotion	Diabetes (Type 2), hypertension, OA, GORD	Daughter (weekly); Son (monthly)
Female R5	86	19	Unspecified	3y, 8 m	Assisted transferring & supervised locomotion	Depression, Hypertension, Sciatica	Daughter (weekly)
Female R6	105	16	Unspecified	7y, 1 m	Mechanically assisted transferring & assisted locomotion	Diabetes (unspecified), renal failure, blindness from glaucoma	Daughter (less than monthly)
Female R7	90	17	AD	11 m	Independent	Paranoid states; OA; Hypertension; Macular degeneration; History of overdose	No visitors (poor relationship with family)
Female R8	86	18	VD	1y, 4 m	Uses walker, needs assistance	NIDDM; OA; Hypertension; CORD	Son & daughter (weekly); Friends & other relatives
Female R9	81	24	AD & VD	1y, 5 m	Uses walker, minimal assistance	Back pain; Dizziness; Hypertension; OA	Daughter (2–3 times/week); Son (weekly); Friends occasionally
Male R10	73	22	AD	2y, 9 m	Recent fall – requires assistance	Falls; depression; Stress incontinence; NIDDM	Wife (weekly); daughter, son, sister, grandchildren (fortnightly)
Female R11	93	22	AD	5y, 6 m	Uses walker, minimal assistance	Neurotic/stress related disorder; Depression; OA	Son & daughter in-law (weekly)
Female R12	87	20	AD	7 m	Independent	OA; Pain; Circulatory disease	Husband (2–3 times/week); Son (weekly)

AD = Alzheimer’s disease.

VD = Vascular dementia.

The three themes that emerged describe QOL as (i) maintaining independence, (ii) having something to do, and (iii) the importance of social interaction and included a number of subthemes (see Table 2). The themes convey not only how QOL is experienced but also the influencers on QOL and how simple changes within facilities can provide the opportunity to improve the QOL of people living with dementia in long-term care.

**Table 2:** **Themes and sub themes**

	**Themes**	**Sub themes/influences**
1.	Quality of life as maintaining independence	i. Structured routine
		ii. Lack of power
		iii. Choice
2.	Having something to do: “You can’t just sit here looking at four walls”	i. Solitary activities
		ii. Organized activities & Outings
3.	The Importance of Social Interaction: “She loves to see people that are from her life”	i. Family
		ii. Residents & staff

### Theme 1: Quality of Life as maintaining independence

Independence was an important element of QOL for most participants. However participants’ views were mixed in regards to whether living in LTC enabled independence. For example, one participant moved into LTC from a retirement village and noted that her QOL, in terms of psychological wellbeing, had improved since the move due to increased independence:

*I didn’t really have much of a quality of life < previously > …because the retirement village… you just fitted in with whatever was happening. You didn’t have much say in anything because it was just organized. But honestly this place is wonderful compared to - well I didn’t know what was going to happen from day to day beforehand… I am able to think for myself instead of, you know, being organized.* (R4)

#### Structured routine

In contrast, other participants found the structured routine of living in LTC challenging. One participant, when asked if there were things she would like to do that she could not do was accepting of the fact that, “*Well you can’t do everything that you want to do when you want to do it*”. (R5) However, she further elaborated that, in contrast to living at home and choosing when she wanted to do things, having a routine in the LTC was challenging.

*Well you fit in, you have to fit in with their… you fit in with their daily, [and] each day is a different day for doing things. You can’t do just exactly what you want because each day is different and they do different things* < to her usual routine >. (R5)

Where participants felt that the facility challenged their independence this negatively affected their QOL. Lack of independence was a very distressing experience for one participant who, when asked how long she has lived in LTC, says, “*It’s too long however long it is. I like my freedom. I want my car and I want to go out and everything*.” (R7) Later she implied that being in LTC was like a prison for her. She further explained the intense distress she felt over being forced to move into the LTC facility and her current sense of feeling trapped there: *Oh yes, it’s … the meanest thing I (sighs)… I’ll never come out of it; it’s here on the book that uh this is permanent.* (R7)

#### Lack of power

Another participant felt that she didn’t have any power to change things within the facility suggesting a lack of control and independence: “*What can I do, I have to be happy right. I have a nice bed. Every thing nice*”. (R8) Her comments suggest a reluctance to share her negative experiences and the sense that she should be grateful for what she has.

While independence and control were not always mentioned directly, the majority of participants shared activities and areas in which they were able to maintain some independence including: feeling a sense of ownership and ability to contribute to the environment through the freedom to create gardens (R8, R10) and having their own belongings and space. (R4)

#### Choice

Participants also valued their ability to have everyday choice in their environment. This ranged from at a more practical level the choice of how the participants could spend their time (R5, R4), to the ability to do their own shopping (R2), and to the choice regarding which facility to move into (R9). More significantly participants talked of the importance of choice in relation to: having own ideas taken into account by staff members. “…*We have a get together sometimes and we are asked to put forward our ideas. I enjoy that*”. (R6)

Choice was quite difficult for some participants as sometimes safety threatened to conflict with participants need for independence. One woman for example was upset when staff wanted to shower her and she explained how she maintained her independence by insisting on showering herself:

*They wanted to shower me. Now I’ve never allowed anyone to shower me. And so they got the message (laughs). Now imagine me at this age having an 18 year old or 20 or 30 shower you. It just wasn’t on. I said nobody showers me, nobody has ever showered me and nobody is ever going to shower me. Well not while I can stand up on my two legs.* (R12)

Another participant who had had a recent hip injury, mentioned that his lawn mower was missing which was something that concerned him and which he was not informed about. He says, “*I had my lawn mower in here but someone flogged (took) it*”. It appeared that staff members removed the lawn mower to prevent him from using it with his current hip injury. The fact that he was not informed suggests a lack of respect for his independence.

### Theme 2: Having something to do: “You can’t just sit here looking at four walls”

Having something to do was an important element of QOL for the majority of participants and was a major influencer on whether participants felt they were able to achieve QOL. However, having something to do did not necessarily mean undertaking activities with others.

#### Solitary activities

Solitary activities were mentioned as being important to approximately half of the participants interviewed including activities such as word finds, knitting and sewing, watching television and movies, and reading books. Several participants mentioned their enjoyment of music. When asked how listening to music made him feel one participant said “*Oh terrific. I mean unless there’s something odd about you… you can’t help but like i*t”. (R3) Another participant, who had not accepted that she lived at the LTC facility, liked playing the piano but did not want to play the piano in the facility. When asked why she said, “*Well it’s not my piano and I think, well some people play it… I’d rather wait until I get home*”. (R12)

There were mixed feelings among the participants about television viewing, which was a common activity in most LTC facilities. For example some participants reported watching television relieved boredom while others were discerning in their television viewing and opted to watch things that interested them and in particular the news. “*I’m not that fussy about TV. I watch it, especially to get the news but TV doesn’t interest me that much. Not that much at all*”. (R6)

Several participants stressed the importance of having quality newspapers to read. When asked what made her feel comfortable in the facility one woman mentioned her subscription to what she regarded as a quality newspaper. She said:

*I subscribe to the Epoch Times… I think it originates from China. It’s a paper that doesn’t go in for ads or anything, it’s just news…It’s just - see air pollution causes cancer so let’s do something about it. And, you know, there’ll be an article on what can be done and what can’t be done. There’s nine commentaries on the Communist Party and what they do and what they shouldn’t be doing (laughs).* (R4)

One man enjoyed doing crosswords from the paper but only if they were “*decent*” - “…*The Sunday papers usually have got one* [a crossword puzzle] *and they’re stupid, they’re not worth doing. If you get decent ones out of the Times…there’s other papers like that, which are very good. Telegraph I think is pretty good and I like doing those but I can’t be bothered with the silly things*” (R3). However, he mentioned that he didn’t receive many newspapers because he’s not seen as intelligent enough - “*They don’t send me an awful lot because they don’t think I’m intelligent*”. (R3)

Several participants regarded walking as an enjoyable activity and a way to avoid idleness and frustration. One participant continued his love of gardening within the facility. He said, “*I like doing things. I do all the gardens around here but that’s come to a sudden end. The way my hip is*”. Other participants were not aware that residents were allowed to garden independently around the facility. When asked if she did any of the gardening she said, “*No… they have their gardeners do it*” (R11). Whereas one woman, who had not accepted that she lives in the facility, talked about enjoying gardening at home but did not want to garden at the facility. She said, “*When I’m home I like gardening*” and later referring to the facility staff she says “…*They wouldn’t want me doing their gardening I wouldn’t think* ”. (R11)

#### Organized activities and outings

The majority of participants felt that participating in certain activities was important for their QOL. One participant said, “*I’m very happy here. All the things that are available, you know… Yes we go out, we have outings. Something’s on every day and all day there’s something on*”. (R4) Another woman said, “…*Well if you didn’t go you’d be sittin’ in your room wouldn’t ya? And you don’t want to do that*”. (R9) Another participant, when asked what made her feel comfortable in the RACF, said “…*I suppose the fact that there’s something on all the time*”. (R4) She also partially attributed her improved health to having things to do - “…*Yes, I’d say my health has improved since I’ve been here… Because there’s activities on. Every day there’s something on*”. (R4)

One participant notably emphasized the importance of activities through sharing the negative effects of not having something to do on her QOL. When asked what sort of things she does during the day she said “*That’s it, idle. Oh I get so frustrated. What can I do? That’s why I walk. I can’t just sit down, I won’t, you know*”. (R7) Although she thought that there were plenty of activities within the RACF she said, “*I don’t like them*”. (R7) Later she described not joining in some activities due to concerns about her age, loss of hearing, and worry about what others might think of her. When asked what QOL is to her she said,

*You haven’t got a life…I’m too old to be sitting in on anything. I’ve had a go with some darn thing and I take too long to sort out exactly what they said. Oh I interrupt that game and if I join in any games you can rest assured they’ll be going tch there she goes again. Oh I can’t hear*. (R7)

The most often-mentioned organized activities were attending church services and going on outings with family members. When asked what it is about the church service that she likes one woman said, “*Well it sort of makes you feel better…can’t explain it but it’s a relief to be well*”. Another man said, “…*I go to church on Sundays and it makes me feel a lot better*”. (R3) Family outings were also an enjoyable activity for many participants. One woman said, “*My daughter’s very, very good. She comes fairly often and takes me for a ride around…Sometimes for lunch. It’s lovely*”. (R2) For some, family outings also served a practical purpose - “…*They take you shopping. If you want anything you go shopping with the family*…”

Several participants noted the role of staff in organizing and facilitating activities. One participant felt that it was important that the staff facilitate activities within the facility so that residents were not watching TV all day - “*It’s always something different that they’re teaching you, you’re not just sitting here watching TV, different days you’re doing something different*”. (R5) Another participant was also appreciative of staff organizing activities and felt it was important to take advantage of the activities - “…*They go to the trouble of, you know, making arrangements for you to go and do things so if you don’t do ‘em they’re not going to help you out either are they*?” (R9)

Several participants’ accounts highlighted that other people should not assume which activities residents will find meaningful. One woman mentioned an ongoing organized activity where school students and residents have an opportunity to meet - the residents visit the school and the students visit the facility. When asked if she enjoys that she said “*Well not particularly to be honest* (laughs*)*”. (R4) Another participant, when asked if he would like to play games like bingo replied, “*No they’re stupid. Some of them are stupid*”. (R3)

In general participants did not think that more organized activities and outings would improve their QOL. Some participants appeared not to have thought about this question. Others seemed resigned to the schedule within the facility. For example when asked whether there are things that could make her feel more content one participant replied “*No I don’t know what you mean by that because everything has to be run in a place like this on a regular, every day has to be different*”. (R5). There were two exceptions with one participant wanting to win the lottery which she imagined would allow her to travel - “*I said to my daughter we’d buy a caravan - she could drive it* (laughs), *we’d go all around Australia”.* (R2)

In general participants focused more on their current abilities than on what they could no longer do. Some expressed gratitude for what they were still able to do. For example one woman was grateful that she could still walk - “…*I love to walk… Whereas some people I feel sorry for…they really go downhill fast a lot of people. And I must thank God that I’m doing as much as I do*”. (R12)

### Theme 3: The importance of social interaction: “She loves to see people that are from her life”

Relationships, conversation and the company of others were very important to most participants. For example, some participants find that friends helped make life meaningful, that talking to people made them happy, and that having company reduced loneliness. When asked what the best thing about her day was one participant replied “*Chatting with other people, hearing their complaints and their worries and trying to give them a little advice, what I’d do if I was in their position and they take it”*. (R6) This example also suggests that altruism can have a positive impact on quality of life.

#### Family

Family was an important source of meaning, enjoyment and support for participants. For many participants QOL was about family: “*Children who are pretty good to you, very good and a good husband. It* [QOL] *would be about them. I absolutely love and adore my son.*” (R12) Another man expressed the pleasure he felt at spending time with his wife when asked about the things in his day that make life meaningful. “*Going out with my wife, I suppose…the pleasure of her company. Yes, it is a pleasure.”* (R1) Another woman expressed the importance of the support her daughter gave her saying “*I’d be lost without my daughter*”. (R2)

Loss of abilities limited the contact some participants had with their family members. For example one participant was unable to go to her grandson’s wedding in London, as she was deemed too old to travel: “…*I can’t get a ticket to go overseas. They say I’m too old to travel. All the family went to the wedding of my grandson in London last year and I couldn’t get a ticket to go. Very sad.”* (R2) This participant also had a son who lived in Russia and who she felt sad not to be able to see. However, her family facilitated her being able to talk with him on Skype on Christmas day: “…*About 9 o’clock we had this computer thing. My son came on from Russia. Oh it was lovely.*” (R2)

Most participants did not mention friends from outside the facility and for many participants memory loss had affected their ability to remember and maintain friendships. For example one man said, “*I don’t think I have any friends that I remember very well at all*”. (R3) Another woman felt very awkward not being able to remember friends who visited her: “*Well I had friends but I had a loss of memory when I came into this place… I’ve visitors that I can’t remember who they are or what their name is - it’s very awkward*”. (R4) There were two exceptions: One participant appreciated that the facility was close to where she used to live as it made it easier for her friends to visit her (R9) and another man said that his wife picks him up and takes him out to visit old friends. (R10)

#### Residents and staff

People within the facility were an important source of company for most participants. Some participants even extended their sense of family to include people in the facility with one participant saying, “*Well we’re all friends, we’re one big family*”. (R2) Other participants like being in the facility as it provides company and reduces loneliness. For example one woman said, “*It’s a wonderful place to be if you’re lonely*” (R2) and another that “…*it’s not as lonely. You’re not home on your own all the time*”. (R9) Like this woman, participants often referred to people in the facility without distinguishing between participants and staff. In these cases it was not clear if they were referring to other participants, staff or both. Another participant didn’t mind staying in the facility and not going out as long as there were others around: “*No I don’t mind staying here. Because there’ll be somebody, as long as there’s somebody else here around. I don’t want to be left in the building by myself*”. (R11)

Other participants were considered an important source of social interaction by approximately half of the participants. When asked what the conversations with staff are like one participant replied, “*Oh well the patients mainly talk to each other*”. (R4) Another participant mentioned friends as an important source of happiness and quality of life for him: “*Talking to people, talking to my neighbors and mates* [are a source of QOL]. (R1)

In sharp contrast to the previous accounts one participant stressed that he enjoys “*intelligent conversation*” and didn’t feel that he was able to have good conversations within the facility: “*I can’t be bothered with… a lot of these fellas here are, they’re short up here I think. I don’t get to have what I call intelligent conversation*”. (R3)

Another participant expressed reluctance to walk around the facility to visit and converse with friends: “You see you talk to the people that you sit at the table with when you go out to meals…” (R5) When asked by the researcher if she was able to get the chance to have conversations with other people in the home she responded: “*Oh yes and no. They couldn’t have people walking around everywhere (laughs)*”. (R5)

Several participants described the loss of friends within the facility. One participant was friends with a woman in the facility for around three years who moved to a different facility nine months ago: “*I did used to have a very nice friend next to me but she didn’t like it here at all…Oh I miss her very much. I was very sad when she left*.” (R2) Another participant, when asked if he had made some mates in the facility, said, “*I have. One of the fellas I’d made a friend, he used to come down to the shed* [men’s shed activity] *with me. Yeah but while I was in hospital this time he died*”. (R10)

Many participants spoke about the staff in very positive terms. One participant said, “*The staff are good, yes. Well, it’s the staff that make the place isn’t it*?” (R1) while another said, “*Oh the carers here, they’re very good. It’s very good here. I can recommend it to anybody*”. (R2) This particular participant also said she has been knitting and sewing Christmas presents for the carers which may be a way of expressing her appreciation. Several participants enjoyed having a joke with the staff and considered the staff as friends, for example, “*Well I think all of them as friends. All the staff are very, very nice and if I can help them, I will, and I don’t put anything in their way so as to make their life difficult for them*.” (R6) This participant also went on to describe the influences of staff on her QOL through mutually respectful relationship that she experienced with staff members.

Despite these positives, most participants felt that staff did not have time for meaningful conversation. For example one participant said, “*Oh well the patients mainly talk to each other (laughs). Yes, because the staff are shifted around, they might be on this floor or they might be over with the mentally handicapped*”. (R4) Another participant, when asked if she was able to have conversations with the staff said, “*Oh yes but they’re all busy*…” (R5) A few participants weren’t interested in talking with staff. For example when asked if she’d rather the staff talk to her more one woman said, “*Oh I don’t care, you know, you come and you go (laughs). I don’t find the time long. You seem to be doing something all the while, you know, you’re coming and going, someone picks you up and puts you down”*. (R9) Staff contact is likely to be more important for participants who lacked social support from family and friends. For example, one participant who has no regular visitors, agreed that it would be good for staff to talk to her more when they came in to do other things such as administer medications. (R7)

## Discussion

This study aimed to understand the perception of QOL reported by older people living with dementia in LTC and more importantly the things that influence their QOL, and the strategies to improve QOL. The three themes that emerged in this study: (i) maintaining independence, (ii) having something to do, and (iii) the importance of social interaction were influenced by the challenges of participants having a progressive cognitive impairment, such as dementia and living in LTC where independence is not readily encouraged, and resident decision-making is actively discouraged. Although long-term care can work well in helping residents with their activities of daily living, the social elements of care such as having something to do and the importance of relationships, conversations and being in the company of others with similar needs and desires did not seem to have a high priority within the study facilities. Participants talked freely of concepts of QOL such as their health (and that of others), and social connectedness and satisfaction as being key influencers on their QOL. They also spoke of the need for acceptance of their living situation as the majority of participants recognised that as a result of increasing frailty and cognitive impairment there was little choice in where they resided. They felt acceptance of their living arrangement would help to improve their QOL and enable them to live a life worth living.

Essential principles of person-centred care are promoted in LTC and are reflected in: the individual having unique value, needs and preferences, the importance of the perspective of the person with dementia allowing freedom of choice and autonomy, the importance of relationships and interactions with others, and physical and emotional comfort that takes into account the needs and preferences of the person with dementia [23]. While person-centred care is advocated as the philosophical tenet or optimal care approach LTC should endorse, the study participants highlight the tensions they experienced of living with other residents in large groups. Person-centred care seemed to be challenged in such situations, especially where residents displayed varying degrees of behavioural symptoms as well as communication abilities, and the LTC approach to care seemed to emphasize safety, efficacy and hierarchical decision-making over individual needs and desires. Importantly participants expressed a strong preference to have some level of control over their routine, as they perceived that the routine structures of the environment seemed to more readily benefit the LTC organization rather than individual residents. Such structures and the lack of meaningful activities are known to diminish QOL for residents with dementia [24].

Internationally long-term care settings are under pressure to maintain a strong model of person-centred care [25], even though it appears that such an approach is not easily maintained in large traditional nursing home environments and where there are strict aged care regulations that providers often interpret as the need for structure and order. Although participants highlight concerns in terms of living with large numbers of residents, when asked what could be done to improve their QOL they did not report the need for major change as most of the time they were satisfied with their situation. However, they expressed the importance of having a space that gave them a sense of ownership. Such a space is more than a physical space that many LTC settings promote as being ‘homelike’, such as having a bedroom decorated with their furniture and photographs – but rather a milieu that promotes the feeling of ownership and where the resident feels they can truly make decisions about things that they feel influence their life.

Participants expressed the importance of their contribution to the LTC environment – they want to make a difference and to feel useful – including advocating simple strategies such as, their involvement in everyday things, for example, maintenance of gardens. Most importantly they want the opportunity to feel they have the power to make change within the environment. Maintenance of independence can be challenging where increasing frailty and diminishing cognition as well as time pressures encourage a situation where staff feel the need to be the decision maker rather than to encourage resident independence. Living in a setting, such as a small group living home, where the daily life is that of a normal household may be one strategy to assist this to occur more readily [26]. Studies have shown the beneficial effects of small group living homes in particular in terms of social engagement, less use of physical restraints [26], the acquisition of a resident role within the environment [27], improvement in QOL [28], and increased attentiveness and responsiveness to resident’s wellbeing [29]. Such facilities are rare in Australia.

The power to make decisions is a challenge for residents, particularly if they have a diagnosis of dementia. The decision-making literature tends to emphasize the role of the health care proxy in treatment and end of life decisions [30], and assessment of patient’s capability to make decisions [31]. The voice of the person with dementia in making everyday decisions seems limited in this literature. Recent research [32] contends that the capacity for people with dementia to be involved in decisions is constrained by under-recognition of their agency. Boyle [32] argues further that a concept of ‘assisted autonomy’ is relevant and needed in supporting people with dementia to be involved in decision making.

People with dementia living in LTC are at risk of isolation and loneliness [33]. Participants in this study highlighted social interaction as an important influence on QOL. While few studies explore social interaction from the perspective of the person with dementia the available literature indicates the importance of social connection to maintenance of wellbeing and prevention of loneliness and social isolation [33,34]. There is the perception that residents in LTC will have other residents and staff to talk to and that as a result relationships will form and will reduce social isolation. While some participants in this study benefited from opportunities to engage in social networks, tensions were also evident as a result of cognitive impairment, for example where families limited their visits and therefore opportunities to network, and where staff had multiple competing obligations that impacted on staff-resident social interaction. Opportunities to engage small groups of residents in conversations have been shown to support resident wellbeing [35]. Prospects to engage residents may be encouraged through consideration of variations in cognitive and physical functioning as well as individual’s behavioural and social functioning in placement /or relocation of residents with similar physical and cognitive abilities [25]. Such opportunities may reduce the perception of participants that the residents did not have the cognitive ability to converse with them.

Our study has limitations and strengths. The small number of participants is a limitation, as is the qualitative nature of the research, which does not allow the data to be generalised. However, the richness of the data comes from the involvement of people with dementia telling their story and their perceptions of their situation. This is strengthened further through the exemplars they provided of their experience. Furthermore, the rigorous approach to training of interviewers, the involvement of three team members in the analysis as well as the qualitative software encouraged research rigor.

## Conclusion

It is commonly assumed that people with dementia are unable to articulate the things that are important to them and that influence their QOL. Our results contribute to the QOL research agenda by showing that the residents in this study were able to discuss important aspects of QOL that need to be solicited, heard and appreciated by care providers and families in order that LTC facilities can become more responsive to factors that support QOL. These findings suggest areas for further research and they have implications for practice and policy. There is a clear need to research further the importance of social connection within LTC and to develop and test interventions that may assist social connection of residents. Furthermore, research that demonstrates an understanding of resident decision-making as well as interventions to assist decision-making is also needed. In addition these findings serve as a caution to LTC providers that rather than just a focus on the physical environment it is imperative that they also consider opportunities to improve the social milieu.

## Abbreviations

AD: Alzheimer’s disease
CORD: Chronic Obstructive Respiratory Disease
GORD: Gastric-Oesophageal Reflux Disease
LTC: Long-term care
MMSE: Mini-mental status examination
NIDDM: Non-Insulin Dependent Diabetes Mellitus
OA: Osteoarthritis
QOL: Quality of life
VD: Vascular dementia
